# From state estimation to active intelligence: safety-aware battery management for electric vehicles

**DOI:** 10.3389/fchem.2026.1960882

**Published:** 2026-09-15

**Authors:** Feng Li, Yuan Li, Wenqi Wu, Huaying Qiao, Lihua Jiang

**Affiliations:** 1 College of Mechanical Engineering, Shandong Huayu University of Technology, Dezhou, Shandong, China; 2 Office of Scientific Research, Shandong Huayu University of Technology, Dezhou, Shandong, China

**Keywords:** battery management system, digital twin, electric vehicle, fast charging, state estimation, state of health, thermal management

## Abstract

Battery management systems (BMSs) are increasingly required to convert incomplete electrical and thermal measurements into safe control actions rather than merely report battery states. This Mini Review focuses on two deployment questions that connect electrochemistry with control: how electrochemical states can be made observable with affordable diagnostics, and how charging, thermal, balancing, and health objectives can be arbitrated when their constraints conflict. We compare physics-based, equivalent-circuit, and data-driven representations using reported quantitative benchmarks, and show why cross-dataset accuracy alone is insufficient evidence of deployability. Particular attention is given to electrochemical impedance spectroscopy (EIS), distribution of relaxation times (DRT), galvanostatic intermittent titration technique (GITT), and active pulse diagnostics as routes for linking measurable responses to interfacial kinetics, resistance growth, and transport limitations. A layered vehicle–edge–cloud architecture is proposed in which a deterministic safety supervisor enforces voltage, temperature, lithium-plating, power, imbalance, and uncertainty limits before performance optimization. The same framework is then examined for solid-state, lithium–sulfur, and sodium-ion batteries, where pressure, observability, and chemistry-specific calibration introduce new constraints. We conclude with a testable short-, mid-, and long-term roadmap centered on uncertainty calibration, pack-level validation, electrochemical observability, version-controlled learning, and safety-case evidence.

## 1 Introduction

The traction battery is no longer a passive energy reservoir. In an electric vehicle, it is a safety-critical electrochemical system whose available energy, power capability, thermal condition, and degradation rate change with temperature, load history, charging behavior, and manufacturing variation. The battery management system (BMS) therefore acts as the operational layer that converts limited measurements—usually terminal voltage, current, and a small number of temperatures—into decisions on power limits, charging, cooling, balancing, isolation, and fault response. The uploaded 2023 review usefully organized this broad field around battery models, state-of-charge (SOC) and state-of-health (SOH) estimation, charging, cell balancing, and thermal management (Ranjith Kumar et al., 2023). Its central message remains valid: reliable management depends on accurate internal-state inference. Yet the research emphasis has shifted. The pressing question is no longer whether model-based or data-driven methods are preferable in isolation, but how they can be combined without sacrificing real-time feasibility, physical credibility, or safety.

Two questions define the focus of this Mini Review. First, what information is actually observable in a vehicle? Voltage-only estimators can be accurate on familiar cells but may become poorly identifiable when temperature, aging mechanism, or chemistry changes (Liu et al., 2026). Electrochemical diagnostics such as EIS and DRT can add mechanistic information, whereas GITT and related pulse tests can improve model parameterization, but their acquisition burden determines whether they are suited to laboratory calibration, periodic service diagnostics, or on-board use. Second, when objectives conflict, which function has authority? A request for maximum charging power at low temperature, for example, should not compete symmetrically with lithium-plating or voltage constraints. Safety-critical coordination requires an explicit hierarchy in which hard constraints veto the performance optimizer.

This review develops a more integrated perspective. Battery models are treated as decision infrastructure rather than stand-alone simulators. State estimation is assessed by its robustness to changing domains, not only by error on a fixed test set. Charging is framed as health- and safety-constrained control, while thermal management and balancing are considered parts of the same pack-level coordination problem. The resulting view is deliberately narrower than a catalogue of algorithms but more relevant to deployment: an effective BMS must know what it does not know, preserve essential physics, exploit field data, and retain a safe fallback when communication or learning components fail.

The integrated framework developed in this review is summarized in Figure 1, where battery representation, state intelligence, charging control, thermal safety, cell balancing, and digital-twin-enabled active sensing are coordinated around the central BMS architecture.

**FIGURE 1:**
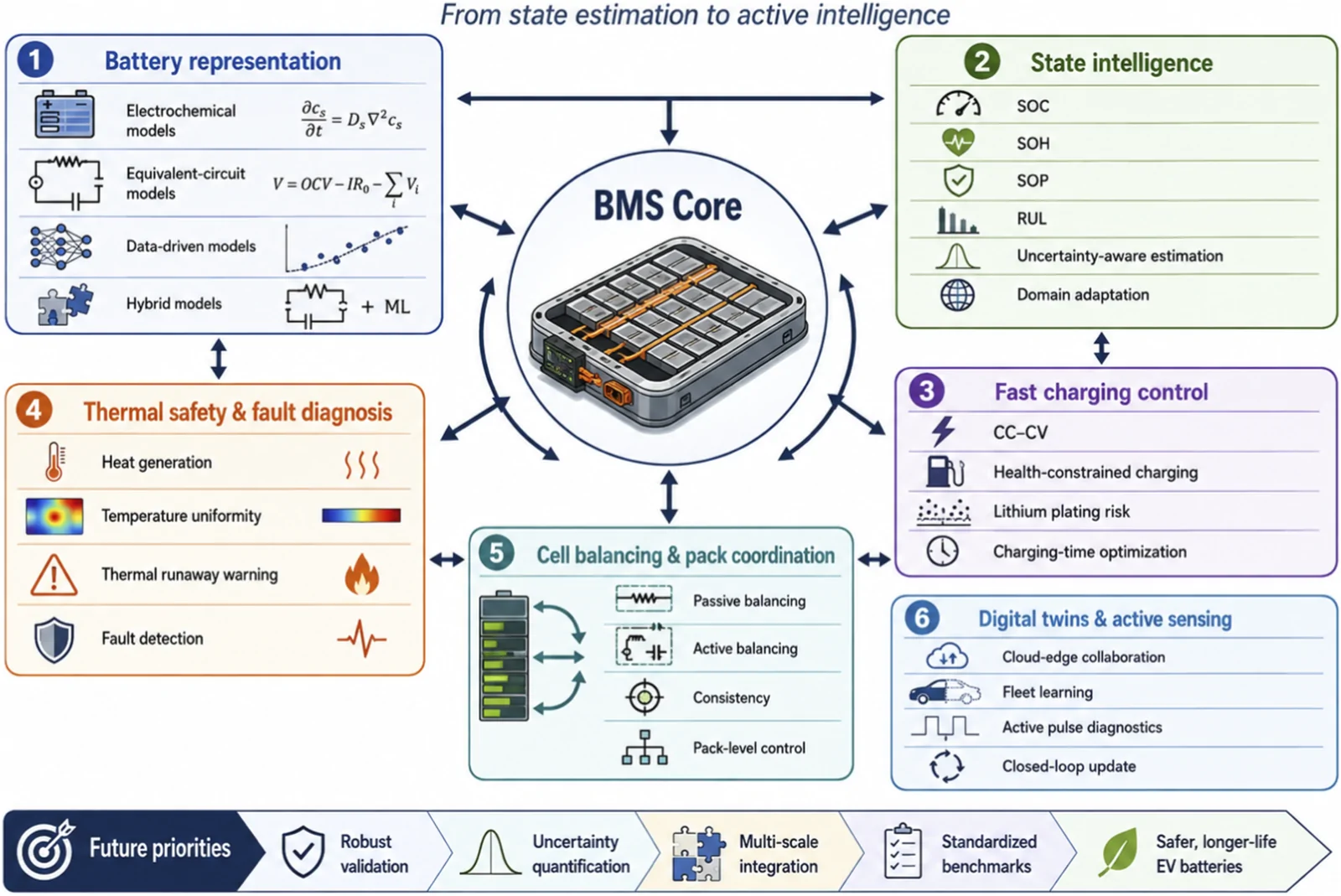
Integrated framework for safety-aware battery management in electric vehicles. Battery representation, state intelligence, fast-charging control, thermal safety and fault diagnosis, cell balancing and pack coordination, and digital twins with active sensing are coupled through the BMS core. Robust validation, uncertainty quantification, multiscale integration, and standardized benchmarks are identified as key priorities for safer and longer-lasting electric-vehicle batteries.

Accordingly, the review is deliberately not an exhaustive catalogue of BMS algorithms. It concentrates on electrochemical observability, uncertainty-aware state intelligence, and constraint arbitration, while treating thermal management, balancing, digital twins, and cloud learning as supporting layers of the same decision system. The vehicle layer operates at millisecond-to-subsecond time scales and retains deterministic protection; the edge layer updates parameters, uncertainty, and diagnostics over seconds to minutes; and the cloud/fleet layer supports slower population learning, model validation, and version-controlled updates. Upward information consists of telemetry, health features, residuals, and uncertainty, while downward information is restricted to validated priors, limits, and policy updates. Hard safety constraints remain local even when connectivity is lost.

Because this Mini Review is intentionally selective, representative studies are used as evidence rather than as an exhaustive bibliography. We prioritize work that contributes at least one deployment-relevant element: mechanistic electrochemical information, a quantitative metric reported with a defined dataset, validation across temperatures/chemistries/domains, module/pack/field evidence, or an explicit statement of computational and sensing limitations. Foundational studies are retained where they define a model or diagnostic concept, whereas recent 2024–2026 work is emphasized when evaluating transfer learning, probabilistic uncertainty, electrochemical diagnostics, physics-informed learning, field-data health estimation, emerging chemistries, and active BMS operation. This evidence hierarchy reduces selective citation and keeps the discussion focused on what can be measured, validated, and safely deployed.

Table 1 summarizes how each functional layer contributes to this architecture and, unlike the previous qualitative summary, now links the stated advantages and limitations to representative evidence. The central argument is that an intelligent BMS should be judged by whether it remains observable, uncertainty-aware, and conservative at safety boundaries—not only by mean prediction error on a familiar test set.

**TABLE 1:** Functional layers of a safety-aware BMS, with explicit information exchange, evidence, and deployment limitations.

Functional layer	Information in/out	Representative approaches	Decision role	Evidence and remaining limitation
Battery representation	V, I, T, operating history → internal states/limits	DFN/reduced electrochemical models; ECMs; hybrid physics–ML	Provides voltage/heat prediction and safety-relevant latent variables	DFN preserves electrochemical causality but is parameter-intensive; ECMs are embedded-friendly but can lose physical uniqueness; model validity shifts with T and aging (Chen et al., 2020; Doyle et al., 1993; Hu et al., 2012; Sulzer et al., 2021)
State and health intelligence	Measurements + parameters → SOC, SOH, SOP, RUL, uncertainty	Adaptive observers, Bayesian filters, GPR/NNs, transfer learning	Converts sparse signals into operational states and confidence	Field labels are limited and domain shift is a dominant failure mode; partial-charge and transfer-learning methods improve practicality but require out-of-domain detection (Deng et al., 2022; Lu et al., 2023; Thelen et al., 2024)
Electrochemical diagnostics	Small perturbations/impedance/relaxation → kinetic and interfacial features	EIS, DRT, GITT, active pulse diagnostics	Improves observability of resistance growth, reaction/transport time scales, and model parameters	EIS/DRT can provide low-error SOH features, but hardware, excitation design, identifiability, and measurement time constrain on-board use; GITT is mainly calibration/service oriented (Khan et al., 2024; LeBel et al., 2022; Su et al., 2024; Zhang et al., 2026)
Fast-charging control	State + constraints + user demand → current/thermal commands	CC–CV, MPC, adaptive/ML-assisted optimization	Optimizes charging only inside an admissible safety set.	Fixed protocols are robust but non-adaptive; aggressive optimization is unsafe if plating, temperature, cell spread, or uncertainty is poorly estimated (Attia et al., 2020; Sun et al., 2024)
Thermal safety and fault diagnosis	T, dT/dt, electrical residuals, gas/pressure where available → hazard probability	Electro-thermal models, multimodal residuals, predictive cooling	Limits peak temperature and non-uniformity; supports early warning	Surface sensing can miss internal hot spots; warning must combine weak signals rather than wait for a single threshold (Bandhauer et al., 2011; Finegan et al., 2015; Gharehghani et al., 2024; Heenan et al., 2023)
Balancing and pack coordination	Cell states + spreads → redistribution and pack limits	Passive/active balancing, pack supervisory control	Prevents weakest-cell constraints from being hidden by pack averages	Voltage equality is not always the correct target when capacity/resistance differ; coordination with charging and cooling is required
Digital twins and active sensing	Life history + model versions + fleet priors ↔ validated updates	Cloud–edge collaboration, digital twins, targeted pulses	Supports longitudinal health tracking and information-seeking diagnostics	Cybersecurity, data ownership, version control, and safe fallback are prerequisites; learning updates cannot bypass the certified local safety envelope (Dubarry et al., 2023; Liang et al., 2026; Wu et al., 2020)

## 2 Battery representation as decision infrastructure

Battery modeling spans a hierarchy from electrochemical descriptions to equivalent-circuit and black-box representations. The Doyle–Fuller–Newman framework resolves lithium transport and reaction processes through coupled partial differential equations and remains the physical reference for many reduced models (Doyle et al., 1993). Such models can expose internal concentration gradients and overpotentials that are directly related to power limitation and lithium-plating risk. Their disadvantage is not simply computational cost. Parameter identifiability, cell-to-cell variability, and uncertain aging mechanisms can make a detailed model appear more precise than the available measurements justify. Parameterization studies and open modeling platforms such as PyBaMM have improved reproducibility, but they also highlight how strongly predictions depend on experimental design and parameter quality (Chen et al., 2020; Sulzer et al., 2021).

Equivalent-circuit models (ECMs) remain attractive for embedded control because a small resistor–capacitor network can reproduce terminal dynamics with modest computation. Comparative work has shown that model order should be chosen for the duty cycle rather than by complexity alone (Hu et al., 2012). The important deployment issue is parameter drift: an ECM calibrated at one temperature and age can fit voltage well while misrepresenting the internal electrochemical margin. GITT-type pulse identification can map OCV and dynamic parameters over SOC and temperature, but the reported pulse durations of roughly 10 s to 30 min and rest periods of about 8 min to 4 h make full characterization unsuitable for continuous on-board operation (LeBel et al., 2022). Thus, high-resolution GITT is best treated as offline or service-level calibration, with only reduced parameter updates performed in the vehicle.

Data-driven models can capture nonlinear residual behavior without specifying every degradation mechanism, but their strongest reported accuracy is often obtained under carefully defined datasets (Severson et al., 2019). This distinction matters because a BMS model is not a leaderboard entry: it must declare its validity domain and provide a safe action when the input leaves that domain. Physics-informed approaches are most useful when the roles are explicit—conservation laws and feasible states are imposed by physics, while learning estimates uncertain parameters or residual effects (Wang et al., 2024). A practical hierarchy is therefore: high-fidelity electrochemistry offline for safety maps and synthetic experiments; reduced electrochemical/ECM models on-board for fast prediction; and learned residuals or parameter adapters for systematic mismatch.

Table 2 addresses the reviewers’ request for quantitative comparison. The numerical results are intentionally reported together with dataset size, chemistry, and validation level because errors from different studies are not directly comparable. For example, an RMSE below 1% on a cell-level laboratory dataset does not imply pack-level accuracy under sensor drift or a new chemistry. Computational cost is therefore expressed as a relative deployment burden when the original paper does not report standardized microcontroller timing; this avoids assigning artificial precision to incomparable hardware.

**TABLE 2:** Representative quantitative benchmarks for battery state/health estimation and their deployment boundaries. Reported metrics come from the cited studies and should not be compared without considering dataset, target, and validation conditions.

Study	Method/target	Reported accuracy	Computational burden*	Sensor/input requirement	Training/chemistry boundary	Validation level	Uncertainty output	Real-time deployability
Roman et al. (2021)	ML pipeline for SOH	RMSE 0.45% (best fast-charge deployment)	Low–medium online; offline training	V/I charge segments	179 Li-ion cells	Cell	Confidence interval reported	Potentially real-time
Deng et al. (2022)	Sparse GPR from random partial charge	Average maximum absolute error 2.88%, 2.52%, 1.51% for 3 cell types	Medium	V/I partial-charge data	Three Li-ion types; multiple T/current conditions	Cell	Predictive variance possible; safety calibration not demonstrated	Yes, if feature window available
Zhu et al. (2022)	Voltage-relaxation ML + transfer	RMSE 1.1% source; <1.7% transfer validation	Low–medium inference	Voltage relaxation after operation	130 cells; NCA, NMC, blended chemistries	Cell	Not primary output	Intermittent; needs relaxation
Ma et al. (2022)	Deep transfer learning	Mean error 0.176% capacity; 8.72% RUL; cross-dataset capacity 0.193%–0.328%	Medium–high training; medium inference	Cycle sensory data	77 cells; >140,000 cycles; cross-scenario/chemistry tests	Cell	Not primary output	Online adaptation demonstrated conceptually
Lu et al. (2023)	Domain-adapted deep SOH model	MAE ≤1.43%; 89.4% samples <3% absolute error without target degradation labels	Medium	Charging/cycling signals	65 cells; 71,588 samples; multiple manufacturers	Cell	No calibrated uncertainty reported	Potentially real-time
Wang et al. (2024)	Physics-informed neural network	MAPE 0.87%	Medium–high training; medium inference	V/I/T and physics constraints	387 batteries; 310,705 samples	Cell	No calibrated uncertainty reported	Embedded feasibility not yet automotive-validated
Khan et al. (2024)	EIS→DRT→LSTM SOH	Average RMSPE 1.69% over ten test sets	Higher acquisition burden	EIS spectrum	5 calendar-aged + 17 cycling-aged Li-ion cells	Cell	No calibrated uncertainty reported	Periodic diagnostic, not continuous
Liu et al. (2025)	NODE–CNN for sodium-ion SOC/SOH	*R* ^2^ = 0.998 SOC; *R* ^2^ = 0.997 SOH	Medium–high training; medium inference	Partial charge + temperature	53 sodium-ion cells, 2 packs, 0 °C–45 °C	Cell + pack	No calibrated uncertainty reported	Promising; pack validation still limited

Accordingly, a deployable comparison should separate estimation error from the conditions required to obtain that error. A method that reports sub-percent RMSE from a controlled charge segment may still be unsuitable for continuous vehicle use if the required segment, rest period, sensor quality, or target-chemistry calibration is unavailable. Conversely, a model with slightly larger nominal error may be preferable if it provides calibrated uncertainty, predictable execution time, and graceful behavior under sensor bias or domain shift. We therefore treat accuracy, acquisition latency, inference burden, validity-domain coverage, uncertainty output, and validation level as co-equal performance dimensions rather than ranking methods by a single error metric.

## 3 State intelligence and electrochemical observability under real-world variability

SOC is often described as a fuel gauge, but unlike liquid fuel it cannot be measured directly. Coulomb counting accumulates sensor bias, open-circuit-voltage methods require sufficient rest, and model-based observers inherit errors in capacity, resistance, and temperature dependence. Reviews and co-estimation studies have therefore emphasized adaptive filtering and joint SOC–SOH estimation (Plett, 2004; Vidal et al., 2020; Xiong et al., 2018; Zou et al., 2015). The practical lesson is that capacity should not remain fixed while SOC is estimated. As the battery ages, an unchanged coulomb-counting denominator produces a systematic SOC bias, particularly near the upper and lower operating limits.

Recent work provides useful quantitative evidence but also illustrates why data context must be reported. Both partial-charging voltage profiles and early-cycle data have been shown to provide informative features for battery health and lifetime prediction (Meng et al., 2019). Roman et al. (2021) obtained an SOH RMSE of 0.45% on 179 cells using charge-curve features and confidence intervals. Deng et al. (2022) used random partial-charging segments and sparse Gaussian-process regression, reporting average maximum absolute errors of 2.88%, 2.52%, and 1.51% for three battery types. Zhu et al. (2022) achieved 1.1% RMSE on a source dataset and below 1.7% after transfer to different commercial chemistries using voltage relaxation. Ma et al. (2022) reported a mean capacity-estimation error of 0.176% on a 77-cell, >140,000-cycle dataset and evaluated transfer across different datasets and chemistry. Lu et al. (2023) further showed that SOH can be transferred without additional target-cell degradation experiments, with MAE ≤1.43% and 89.4% of samples below 3% absolute error. These results are strong evidence for feature and transfer design, but they do not remove the need to detect when a vehicle has left the training domain (Lanubile et al., 2024; Tao et al., 2024).

Field data make this problem harder because availability and informativeness are different. Real vehicles provide large volumes of voltage/current records but often contain narrow SOC windows, uncertain calibration, firmware changes, missing rest periods, and no direct capacity labels. Driving-behavior-guided feature screening explicitly evaluates whether candidate health indicators are observable under real driving (Jiang et al., 2024), while multimodal field-data frameworks attempt to combine complementary signals (Liu et al., 2025). A production estimator should consequently output three items: an estimate, calibrated uncertainty, and a validity flag linked to domain coverage. Validation should deliberately inject current/voltage bias, missing sensors, ambient-temperature shifts, replacement modules, and abnormal but non-fault conditions rather than relying only on random train–test partitions (Thelen et al., 2024).

### 3.1 Electrochemical diagnostics: EIS, DRT, GITT, and active pulses

The electrochemical emphasis can be strengthened by distinguishing diagnostics according to the physical information they add. EIS perturbs the cell over frequency and separates processes that are compressed into a terminal-voltage transient. Its BMS value is not the Nyquist plot itself but access to features associated with ohmic resistance, charge transfer, diffusion, temperature, and interphase evolution. Recent reviews emphasize progress in miniaturized impedance measurement, rapid extraction, validation, and interpretation for battery safety applications (Wen et al., 2025; Zhang et al., 2026). However, a full laboratory spectrum is not automatically an on-board measurement: excitation amplitude, frequency selection, power-electronics noise, DC bias, and measurement time must be designed around vehicle constraints.

DRT is useful because it maps an impedance spectrum into characteristic relaxation times without requiring the reviewer or algorithm to preselect a specific ECM topology. Su et al. (2024) developed DRT-based features and reported SOH RMSE within 0.873%. Khan et al. (2024) applied DRT to EIS from five calendar-aged and 17 cycling-aged cells and obtained an average RMSPE of 1.69% across ten test sets using an LSTM estimator. Zhao et al. (2024) similarly used DRT to separate overlapping impedance contributions before data-driven SOH estimation. These studies show that electrochemical features can be quantitatively competitive; the remaining step is pack-level acquisition with sparse frequencies or targeted excitation, uncertainty propagation, and validation under converter noise.

GITT occupies a different role. It can identify OCV and dynamic ECM parameters over SOC and temperature with high resolution, but long pulse/rest sequences make it more appropriate for laboratory characterization, manufacturing calibration, or service diagnostics than continuous vehicle control (LeBel et al., 2022). Active pulse diagnostics provide a bridge: instead of sweeping a full spectrum or waiting for long rest, the BMS inserts short bounded excitations during suitable operating windows and extracts information-dense dynamic features. The active-BMS concept therefore turns excitation into part of sensing (Liang et al., 2026). Such pulses must remain within electrochemical and comfort constraints and must be separated from power-electronics disturbances.

These diagnostic tools should therefore be viewed as a hierarchy rather than substitutes. Full-spectrum EIS is most informative when interfacial and transport processes must be separated; DRT converts that spectrum into time-scale features that can support mechanism-aware SOH estimation; GITT provides high-resolution OCV and dynamic parameter surfaces but at an acquisition cost that generally confines it to laboratory, manufacturing, or service calibration; and short bounded pulses offer the most practical route to on-board information seeking when uncertainty becomes high. The central electrochemical design question is thus not which diagnostic is universally best, but what minimum excitation can resolve the safety-relevant process of interest within the available time, power-electronics noise, and aging budget (Khan et al., 2024; Liang et al., 2026; Su et al., 2024; Wen et al., 2025; Zhang et al., 2026).

As summarized in Table 3, electrochemical diagnostic methods provide complementary information on battery health, internal kinetics, and degradation, supporting accurate state estimation and safety-aware decision-making in a deployable BMS (Birkl et al., 2017).

**TABLE 3:** Electrochemical diagnostic methods and their roles in a deployable BMS.

Method	Electrochemical information	Representative evidence	Acquisition/interpretation burden	Recommended BMS role
EIS	Frequency-dependent impedance; ohmic, charge-transfer, diffusion and thermal/aging signatures	Supports SOC/SOH/temperature and fault features; recent work targets miniaturized on-board impedance hardware (Wen et al., 2025; Zhang et al., 2026)	Full spectra require perturbation design and are vulnerable to DC bias, ripple, wiring and converter noise	Periodic on-board or service diagnostics; sparse-frequency implementations are most practical
DRT applied to EIS	Characteristic time-scale distribution without fixing a single ECM topology	SOH RMSE ≤0.873% in Su et al. (2024); average RMSPE 1.69% in Khan et al. (2024)	Ill-posed inversion needs regularization; peak interpretation and uncertainty can change with temperature/chemistry	Feature extraction and mechanistic health indicators from EIS.
GITT/pulse parameterization	OCV and dynamic response versus SOC/T; diffusion/kinetic parameter support	High-resolution ECM response surfaces; LeBel et al. (2022) used >120 titration points over −20 °C to 60 °C	Typical pulse/rest protocols are long (seconds–minutes pulses; minutes–hours rest)	Offline characterization, calibration, service-level diagnostics
Active bounded pulses	Short dynamic response under intentionally selected excitation	Active BMS uses information-seeking pulses to improve observability (Liang et al., 2026)	Pulse must be safe, imperceptible, and identifiable from inverter/charger disturbances	On-board targeted diagnosis triggered by high uncertainty or weak observability

## 4 Fast charging as explicit constraint arbitration

Fast charging is commonly presented as a trade-off between charging time and battery life. In practice it is a multi-constraint control problem. High current increases ohmic and reaction heat, while low temperature or high SOC can promote lithium plating because anode transport and intercalation cannot accommodate the imposed flux. Cell inconsistency means that the most vulnerable cell, rather than pack-average SOC, sets the safe current. A charging protocol must therefore coordinate voltage limits, temperature, estimated anode risk, cooling capability, and the spread of cell states.

Traditional constant-current/constant-voltage charging remains attractive because it is simple, certifiable, and robust. Its limitation is that the same current schedule is applied despite differences in age, temperature, and recent use. Closed-loop experimental optimization demonstrated that machine learning can search a large protocol space using early-cycle feedback rather than exhaustive lifetime testing (Attia et al., 2020). More recent work treats artificial intelligence as a tool for life-cycle adaptation, combining charging data, degradation prediction, and control constraints instead of optimizing a single fixed protocol (Sun et al., 2024).

A practical arbitration rule is to compute independent admissible current limits for terminal voltage, temperature, plating risk, pack power, cell imbalance, and uncertainty. The performance optimizer—whether MPC, dynamic programming, or a learned policy—operates only inside this feasible set. This creates a lexicographic priority: (1) hard safety and isolation, (2) lithium-plating and cell-voltage margin, (3) thermal envelope, (4) degradation and balancing objectives, and only then (5) charging-time demand. If sensor validity or uncertainty fails a threshold, the supervisor derates current or returns to a validated conservative profile rather than extrapolating the optimizer.

Figure 2 makes this rule operational for low-temperature fast charging. The request is first evaluated using SOC, SOH, cell temperatures, cell spread, uncertainty, and recent history. A thermal gate can require preheating and current derating before the electrochemical and pack gates are evaluated. The controller then calculates the current limits and optimizes charge time, aging, and thermal uniformity only after the safety supervisor has defined the feasible current. This resolves the reviewer’s example directly: when a fast-charge request conflicts with a cold-cell plating or thermal constraint, the user-performance objective is subordinated to the electrochemical safety envelope.

**FIGURE 2:**
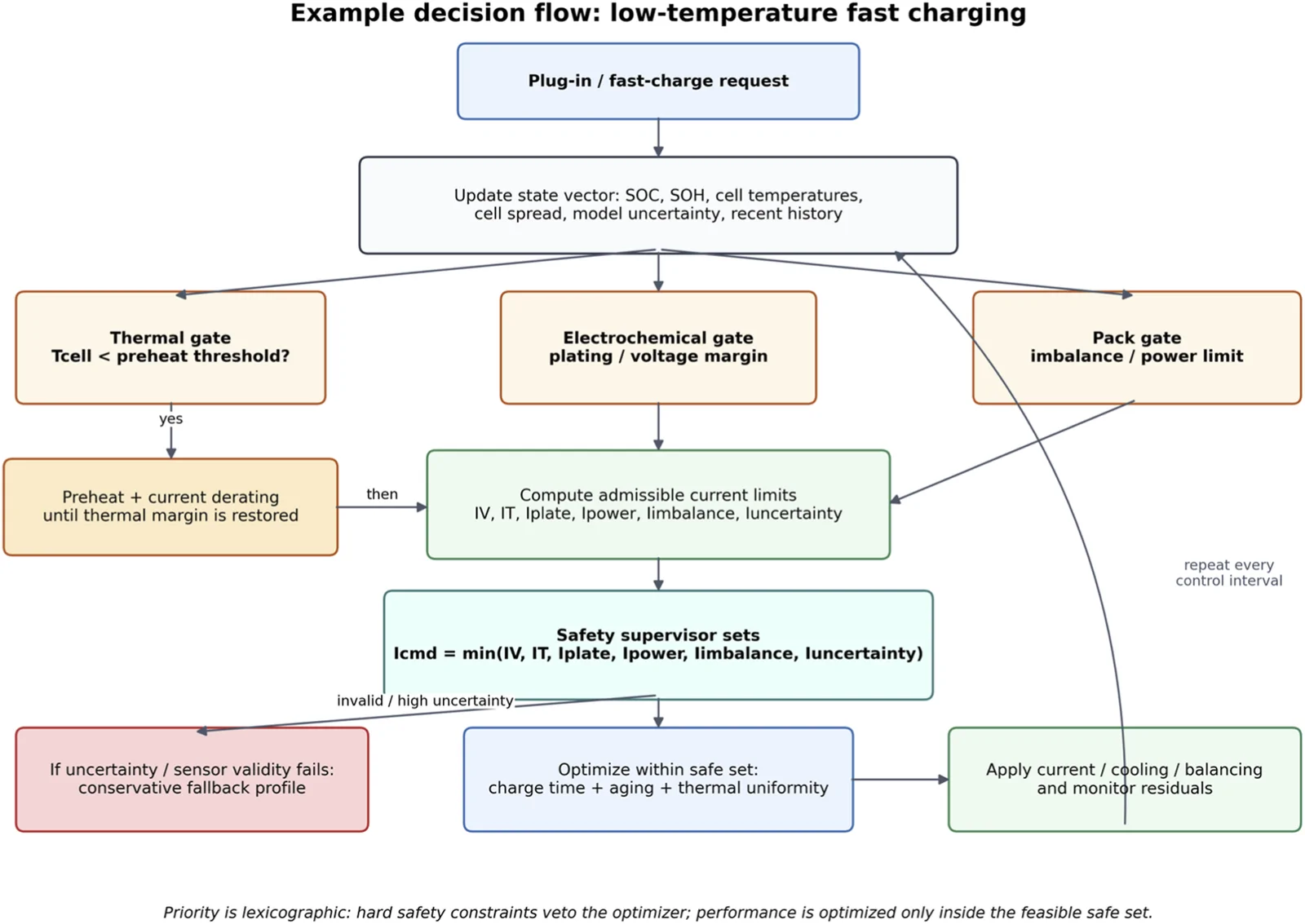
Decision flow for a low-temperature fast-charging request. State/health/uncertainty estimates feed thermal, electrochemical, and pack gates; the safety supervisor computes the most restrictive admissible current, while charge-time optimization is performed only within that safe set. Invalid sensing or high uncertainty forces a conservative fallback.

Cloud prediction can still be useful without becoming part of the hard safety loop. For example, Li et al. (2025) trained a model on 909,135 real-world direct-current charging sessions and reported 90% charging-duration accuracy from one observation and 95% accuracy with absolute error below 1 min using six observations within 5 min. Such predictions can support station scheduling, thermal preconditioning, and driver information. They should not directly override cell-level voltage, temperature, plating, or uncertainty limits because prediction of user-visible charging duration is a different task from certifiable safety control.

## 5 Thermal safety, fault detection, and cell balancing as pack-level constraints

Temperature is both a state and a source of estimation error. Surface sensors provide a sparse, filtered view of internal heat generation. Operando measurements have demonstrated how strongly internal temperature depends on cell design and rate: in the study of Heenan et al. (2023), a 20 min high-rate discharge of an energy-optimized 3.5 Ah cell produced internal temperatures above 70 °C, whereas a power-optimized design under a shorter high-rate condition remained below about 50 °C; even under the same 6 A current, internal thermal responses differed substantially. The lesson for BMS design is not to transplant those exact temperatures to every pack, but to recognize that pack-average surface temperature can hide chemistry- and design-dependent internal hot spots.

Air, liquid, refrigerant, heat-pipe, and phase-change systems impose different mass, hydraulic, fault-tolerance, and controllability constraints, and current reviews increasingly emphasize hybrid thermal architectures (Gharehghani et al., 2024). The BMS should use this hardware predictively. Before cold fast charging, preheating can increase the electrochemical current margin; during high load, coolant flow can be allocated according to predicted cell heat and cell-to-cell spread; and after operation, delayed heat release should be considered before parking or immediate recharge. The thermal objective is therefore two-dimensional: constrain both the maximum temperature and spatial non-uniformity.

Thermal runaway further illustrates why detection and mitigation cannot be separated. High-speed imaging has revealed rapid internal structural changes after runaway onset (Finegan et al., 2015), while electro-thermal analyses show the positive feedback among heat generation, side reactions, and heat transfer (Bandhauer et al., 2011). A practical warning layer should fuse weak evidence—voltage divergence, dT/dt, impedance change, gas/pressure where available, and residual inconsistency between electrical and thermal models—into a calibrated hazard probability. A single high-temperature threshold is simple to certify but often too late; an opaque classifier with no uncertainty is earlier but difficult to trust. The appropriate architecture combines interpretable residuals, probabilistic confidence, and deterministic escalation actions.

Balancing is part of this same constraint system because the weakest cell determines end-of-charge margin and fast-charge current. Passive balancing is predictable but dissipates heat; active balancing can redistribute energy but adds converter loss, hardware faults, and supervisory complexity. Equal terminal voltage is not necessarily the correct target when cells have different capacities and resistances. Depending on the operating objective, the pack may instead minimize predicted end-of-charge margin, SOC spread, heat-generation spread, or future power limitation. Charging, cooling, and balancing should therefore share the same cell-level state and constraint estimates rather than optimize separate surrogate objectives.

## 6 Digital twins, active sensing, chemistry transfer, and safety assurance

A battery digital twin is most useful when it maintains identity, parameter history, uncertainty, and traceable links among cell, module, pack, vehicle, and fleet information rather than serving as a dashboard or duplicate neural network (Dubarry et al., 2023; Wu et al., 2020). The cloud can improve long-horizon priors and fleet statistics, the edge layer can adapt parameters and select validated policies, and the vehicle retains deterministic protection and short-horizon control. Digital-twin-supported transfer learning illustrates how continuously updated data models can support state estimation (Zhao et al., 2025), but each update needs model identity, training-data lineage, validation results, and rollback capability.

Active sensing closes the observability loop. Instead of waiting for normal driving to excite every relevant dynamic mode, the BMS can schedule a bounded pulse, altered charging segment, or short rest when uncertainty becomes high. The resulting measurement updates the state/model and may alter the next control decision (Liang et al., 2026). This is a genuine transition from passive estimation to active intelligence because the controller chooses not only an action but also an information-gathering experiment. The safety condition is that the experiment itself remains inside a validated electrochemical and thermal envelope.

### 6.1 Emerging chemistries: what is transferable and what must be recalibrated

The architecture can be chemistry-agnostic at the level of information flow—sensing, estimation, uncertainty, constraint supervision, actuation, and version-controlled learning—but its state definitions, constraints, and diagnostic maps are chemistry-specific. For all-solid-state batteries, stack pressure becomes a coupled state/control variable because contact loss, interfacial impedance, cracking, and short-circuit risk depend on electro-chemo-mechanical conditions. Reviews emphasize that many laboratory solid-state cells still rely on high external pressure, whereas practical targets are moving toward much lower operating pressure; Xu et al. (2024) note industrial requirements below approximately 1 MPa, and recent low-pressure reviews identify pressure reduction as a central commercialization problem (Zhang et al., 2025). A solid-state BMS may therefore need pressure or force sensing, pressure-dependent impedance models, and diagnostics capable of distinguishing contact loss from ordinary electrochemical aging (Biçer et al., 2025).

Lithium–sulfur cells illustrate a different observability problem. Their voltage behavior contains regions in which conventional OCV and Coulomb-counting assumptions are less informative, and state estimation often requires chemistry-specific dynamic or electrochemical models. Reviews of Li–S BMS methods therefore emphasize Bayesian filtering and data-driven alternatives tailored to sulfur reaction dynamics rather than direct reuse of lithium-ion observers (Ayadi et al., 2022). For sodium-ion batteries, much of the BMS architecture is transferable, but OCV–SOC relations, thermal behavior, aging signatures, and safety limits require recalibration (Sandri et al., 2026). Encouragingly, Liu et al. (2025) reported R^2^ = 0.998 for SOC and 0.997 for SOH using partial charging data across 53 sodium-ion cells and two packs at 0 °C–45 °C. This result supports transfer of methodology, not transfer of parameters.

Pack-level validation should therefore report coupled outcomes rather than isolated estimator accuracy. At minimum, a stress test should record the maximum cell temperature, cell-to-cell temperature spread, voltage/SOC spread, minimum electrochemical safety margin, false-alarm and missed-detection rates for fault logic, 95th-percentile or worst-case state-estimation error near operating limits, and end-to-end control latency. The same protocol should be repeated under sensor bias, missing measurements, cooling degradation, aged-cell mismatch, and communication loss. Such reporting reveals whether coordination actually protects the weakest cell, rather than merely improving an average pack metric.

The transferable/non-transferable boundary can therefore be stated explicitly. The architecture of sensing → estimation → uncertainty assessment → constraint supervision → actuation → version-controlled learning can remain chemistry-agnostic. In contrast, the latent states to be estimated, the electrochemical features that are observable, the safe voltage/temperature/pressure envelope, degradation signatures, and the mapping from diagnostic features to failure modes must be recalibrated for each chemistry and cell design. This distinction is essential for avoiding negative transfer: a common software architecture can reduce development burden, but common numerical parameters or diagnostic thresholds should not be assumed across lithium-ion, solid-state, lithium–sulfur, and sodium-ion systems.

### 6.2 Safety certification and continuously learning BMS functions

Learning-enabled BMS functions also need an assurance architecture. ISO 26262 applies functional-safety lifecycle principles to safety-related electrical/electronic systems in series-production road vehicles. It does not provide a ready-made certification recipe for every ML estimator, but it motivates traceable requirements, hazard analysis, verification evidence, and controlled change. UL 4600 is an autonomous-products safety standard rather than a battery-specific BMS standard; nevertheless, its safety-case philosophy is relevant when AI-enabled supervisory functions contribute to a vehicle-level safety argument (UL Standards and Engagement, 2023). These standards should therefore be cited with their actual scope rather than presented as direct certification standards for battery algorithms.

A continuously learning controller creates a specific difficulty: the deployed function changes after validation. A safer near-term pattern is to freeze the safety-critical on-board model version, run new learning components in shadow mode or at the edge/cloud, validate them against a predefined test matrix, and deploy only signed versioned updates that retain rollback. Validation should include nominal accuracy, 95th-percentile or worst-case error near safety limits, uncertainty calibration, sensor faults, domain shifts, adversarial/corrupted updates, loss of connectivity, and timing/memory constraints. The deterministic safety supervisor in Figure 1 provides a stable boundary around adaptive functions, allowing learning to improve performance without becoming the sole barrier against electrochemical abuse.

A practical assurance chain for a new learning component is therefore: offline training with traceable data and fixed preprocessing; shadow-mode evaluation without control authority; regression, fault-injection, and domain-shift testing; hardware-in-the-loop verification of timing and fallback behavior; limited vehicle validation; signed release with a unique model version; and rollback if post-deployment monitoring detects performance outside the approved envelope. Continuously learning functions may update fleet priors or candidate models in the cloud, but a safety-critical on-board estimator or controller should not change autonomously between validated releases. This separation makes the learning process compatible with a stable safety case and preserves reproducibility after software updates.

## 7 Actionable research priorities and conclusion

The research priorities can now be stated as testable engineering programs rather than general calls for ‘better data’ or ‘better models’. Table 4 organizes them by horizon, technology-readiness target, required evidence, and dominant failure mode. In the short term, the field needs common reporting of domain coverage, uncertainty calibration, computational burden, and boundary-case error. In the mid term, the central experiment is pack-level closed-loop arbitration under combined stressors—cold fast charging, cell imbalance, sensor bias, and thermal non-uniformity—using hardware-in-the-loop and vehicle tests. In the long term, chemistry-adaptive digital twins and active diagnostics should be evaluated through traceable safety cases in which every model update has data lineage, version identity, validation evidence, and rollback.

**TABLE 4:** Proposed testable roadmap for safety-aware active BMS development. TRL ranges are proposed targets rather than ratings of the cited literature.

Horizon	Specific objective	Measurable deliverable	Data requirement	Validation/target TRL	Major failure modes
Short term (1–3 years)	Electrochemical observability + uncertainty	Report boundary-case error, calibration curves, runtime/memory; compare sparse EIS/DRT, relaxation and pulse features under common protocols	Multi-temperature cell datasets with sensor bias, missing data, multiple aging modes, raw diagnostic metadata	Cell + HIL; target TRL 4–5	Domain shift; false confidence; diagnostic noise
Short term (1–3 years)	Reproducible model cards	Record chemistry, format, current/T range, sampling, preprocessing, target labels, compute platform and known invalid regions	Public benchmark splits and independent hold-out cells	Cell/module replication; target TRL 4–5	Selective reporting; hidden leakage; incomparable metrics
Mid term (3–5 years)	Closed-loop conflict arbitration	Demonstrate hard-constraint veto and graceful derating during cold fast charge, imbalance, cooling limits and sensor faults	Pack datasets with synchronized electrical/thermal signals and controlled fault injection	Module/pack HIL + vehicle; target TRL 5–6	Weakest-cell violation; unstable optimizer; sensor drift
Mid term (3–5 years)	Pack-level active diagnostics	Trigger information-seeking pulses only when expected information gain justifies electrochemical/comfort cost	Pack impedance/dynamic data with converter noise and repeated aging	Pack/vehicle; target TRL 5–6	Unidentifiable pulse response; extra aging; EMI contamination
Long term (5 + y)	Chemistry-adaptive digital twin	Transfer architecture while recalibrating chemistry-specific observability and constraints; include pressure for solid state	Cross-chemistry fleet datasets with traceable versions and environmental metadata	Fleet/vehicle; target TRL 6–8	Negative transfer; pressure/contact failure; new aging modes
Long term (5 + y)	Safety case for learning updates	Signed frozen releases, shadow-mode evaluation, rollback, cybersecurity and evidence-based approval gates	Longitudinal field evidence + regression/fault/adversarial test suites	Vehicle/fleet safety case; target TRL 6–8	Unsafe update; corrupted data; connectivity loss; certification drift

Standardization is essential because a model that uses a complete charge curve should not be compared directly with one restricted to real-time signals, and a cell-level laboratory benchmark is not equivalent to a pack-level field trial. Future publications should therefore state whether target-battery calibration data were used, whether uncertainty was calibrated rather than merely produced, and whether reported latency includes feature acquisition. For electrochemical diagnostics, the acquisition protocol—frequency set, perturbation amplitude, SOC, temperature, and validation criteria—should be reported together with the state-estimation metric.

These priorities should be judged against explicit acceptance evidence. In the short term, a study should report boundary-case error, uncertainty calibration, acquisition time, inference runtime/memory, and failure regions on independent cells. In the mid term, coordinated control should demonstrate that hard-constraint veto and graceful derating remain stable under combined cold charging, imbalance, sensor faults, and thermal non-uniformity at module/pack level. In the long term, chemistry-adaptive and learning-enabled BMS functions should provide versioned data/model lineage, cross-chemistry validation, cybersecurity testing, signed updates, and rollback evidence at vehicle/fleet scale. A research priority is therefore considered mature only when its claimed benefit remains valid under the failure mode that is most likely to invalidate it.

Battery management is becoming coordinated intelligence, but the coordination must be explicit. Physics-based models preserve causal constraints; ECMs provide embedded speed; data-driven models capture residual variability; electrochemical diagnostics improve observability; and digital twins connect information across time and scale. None should be granted authority outside its validity domain. The most credible route to safer and longer-lived electric-vehicle batteries is therefore a layered BMS in which uncertainty-aware estimation and electrochemical diagnostics define what is known, a deterministic supervisor defines what is allowed, and adaptive optimization determines what is preferred only inside that safe set. This principle remains transferable to emerging chemistries, while the parameters, observability maps, and constraints must be recalibrated for each electrochemical system.
